# Large Osteophytes over 10 mm at Posterior Medial Femoral Condyle Can Lead to Asymmetric Extension Gap Following Bony Resection in Robotic Arm–Assisted Total Knee Arthroplasty with Pre-Resection Gap Balancing

**DOI:** 10.3390/jcm12185980

**Published:** 2023-09-15

**Authors:** Jong Hwa Lee, Ho Jung Jung, Joon Kyu Lee, Ji Hyo Hwang, Joong Il Kim

**Affiliations:** 1 Department of Orthopaedic Surgery, Kangnam Sacred Heart Hospital, Hallym University College of Medicine, Seoul 07441, Republic of Korea; bigdawg@hallym.or.kr (J.H.L.); hwangjihyo7309@gmail.com (J.H.H.); 2 Department of Orthopaedic Surgery, Chuncheon Sacred Heart Hospital, Hallym University College of Medicine, Chuncheon 24253, Republic of Korea; hodge.jung@gmail.com; 3 Department of Orthopaedic Surgery, Konkuk University Medical Center, Konkuk University School of Medicine, Seoul 05030, Republic of Korea; ndfi@naver.com

**Keywords:** robotic arm–assisted total knee arthroplasty, total knee arthroplasty, osteophytes, gap balancing

## Abstract

Robotic arm–assisted total knee arthroplasty (TKA) involves a pre-resection gap balancing technique to obtain the desired gap. However, the expected gap may change owing to the soft-tissue release effect of unreachable osteophytes. This study evaluated the effect of unreachable osteophytes of the posterior medial femoral condyle on gap changes following bony resection. We retrospectively analysed 129 robotic arm–assisted TKAs performed for varus knee osteoarthritis. Knees were classified according to the size of osteophytes on the posterior medial femoral condyle using preoperative computed tomography measurement. After the removal of reachable osteophytes, the robotic system measured pre- and post-resection medial extension (ME), lateral extension (LE), medial flexion (MF), and lateral flexion (LF) gaps. No extension gap changes were observed for 25 (19.4%), and no flexion gap changes were observed 41 (31.8%) knees, following bone cuts. ME, LE, MF, and LF gaps increased with the osteophyte size (*p* < 0.05). For osteophytes <10 mm, all the gaps increased symmetrically. However, for osteophytes >10 mm, the ME gap increased asymmetrically more than LE, MF, and LF gaps (*p* < 0.05). The gap changes due to bony resection were correlated to the osteophyte sizes of the posterior medial femoral condyle. Surgeons should plan a slightly tight medial extension gap to attain the desired gaps for >10 mm osteophytes.

## 1. Introduction

Precise extension and flexion gap balancing represent critical objectives in achieving favourable short- and long-term outcomes for total knee arthroplasty (TKA) [1]. Traditionally, proper soft-tissue balance has been characterised by equivalent and symmetrical flexion and extension gaps [2,3]. Studies have demonstrated that attaining this balance can effectively reduce postoperative instability and stiffness, thereby decreasing the need for revision surgeries and substantially enhancing patient-reported outcomes [4,5].

Robotic arm–assisted TKA (R-TKA) was developed to increase the accuracy of gap balancing and bone cutting through the incorporation of preoperative computed tomography (CT) planning and intraoperative kinematic data [6]. Furthermore, the final ligament balance can be objectively quantified using the robot-assisted system [7]. Since the introduction of these systems, multiple studies have shown that certain robotic arm–assisted systems are more accurate and efficient at balancing gaps than manual systems [8,9,10]. 

In R-TKA, following the removal of reachable osteophytes and preliminary soft-tissue release, pre-resection gap balancing is performed by modifying the implant position according to the intraoperative gaps verified by the robot system. However, once balanced, these gaps can change following bone resection. Cutting of the femoral and tibial bones can loosen the collateral ligament and joint capsule, and unreachable osteophytes of the posterior femur that cannot be completely removed before bone cutting may affect gaps by eliminating the tenting effect [11,12]. If the gap increases asymmetrically following bone cuts, additional procedures for ligament balancing and bone recutting must be performed to match the mediolateral flexion–extension gaps. These issues negate the advantages of robotic systems, which emphasise precise bone cutting and reduced soft-tissue release [13].

The primary objectives of this study were to (1) quantitatively assess the accuracy of predicting the post-resection gap in R-TKA and (2) investigate the influence of unreachable osteophytes of the posterior medial femoral condyle, with specific reference to changes in gaps following bone resection. We hypothesised that there would be a discernible difference between the predicted and actual post-resection gaps and that this difference would be more pronounced in patients with larger osteophytes. The results of this study should provide valuable insights into improving soft-tissue balance during TKA procedures and contribute to enhancing the overall success and outcomes of surgical intervention.

## 2. Materials and Methods

### 2.1. Patients

In this analysis, 129 varus knees that underwent TKA for primary osteoarthritis between November 2019 and February 2023 were included. Patients with a history of femoral or tibial fractures, valgus knee deformity, osteotomy, rheumatoid arthritis, post-traumatic arthritis, or pyogenic arthritis of the knee joints were excluded. For the TKA procedures, posterior-stabilising prostheses (Triathlon^®^; Stryker, Kalamazoo, MI, USA) were implanted using the MAKO Robotic Arm Interactive Orthopaedic System (Stryker, Kalamazoo, MI, USA). The robotic system enabled the precise measurement of the medial and lateral gaps in flexion and extension before and after femoral bone cutting. The study protocol was approved by the institutional review board.

### 2.2. Surgical Techniques

All surgical procedures were performed by a single experienced surgeon (J.I.K.) using the medial parapatellar approach. Two pins were inserted into the femur and tibia and positioned approximately 10 cm from the main skin incision, and the femoral and tibial sensor arrays were then fixed onto the pins. Patient-specific CT-based bone models were confirmed using registered landmarks, and kinematic data were integrated to adjust the preoperative plan based on CT scans, with the goal of achieving optimal knee balance. After removing the anterior and posterior cruciate ligaments and accessible femoral osteophytes, the extension gap at 10° of flexion, or up to 25° if a flexion contracture was present, and the flexion gap at 90° of flexion were recorded with the robot system. Maximal manual varus and valgus stresses were applied to tension the collateral ligaments in the knee extension state to measure pre-resection extension mediolateral gaps. Gaps at 90° of flexion were measured using maximal-size spacer spoons in the medial and lateral compartments (gap in planning). Following the distal, anterior, and posterior femoral and tibial bone cuts, the trial components were inserted, and the resulting extension mediolateral gaps and flexion mediolateral gaps were recorded with the robot system (gap after cutting).

### 2.3. Radiographic Measurement

Leie et al. [11] devised an assessment of osteophytes of the posterior femoral condyle using plain radiographs and classified them into four different categories. As all patients who undergo R-TKA require CT scans for preoperative planning, we utilised the method described by Leie et al. to measure the size of the osteophytes using CT scans for optimal accuracy. The sagittal view of the CT scan was carefully assessed to obtain measurements of osteophyte size. Since the size of the osteophytes on the medial femoral condyle was thicker than on the lateral femoral condyle in all cases, we assumed that assessing the size of the osteophytes on the posterior medial femoral condyle reflected the overall amount of osteophyte.

**Figure 1:**
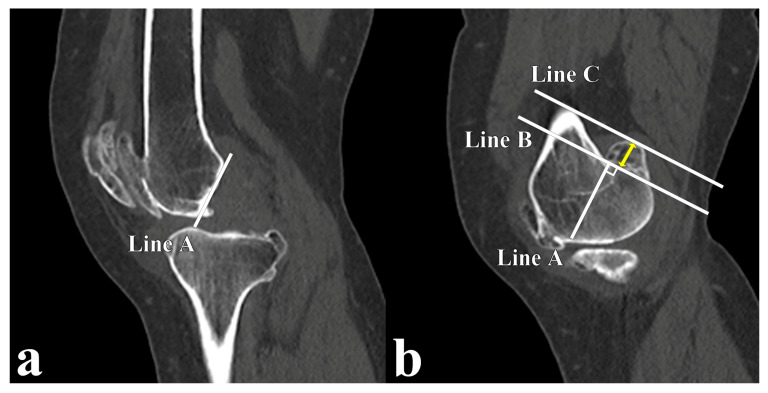
Technique of osteophyte size measurement. (**a**) Midsagittal section of CT: A line is drawn on the Blumensaat line extending from the anterior to the posterior-most aspect of the femoral cortex (Line A). (**b**) Sagittal section of CT with largest osteophytes: Line A is copied to sagittal section of CT with largest osteophytes, and second line (Line B) is drawn on the most posterior aspect of femoral cortex, perpendicular to Line A. A third line (Line C) is drawn parallel to line B and on the most posterior edge of osteophyte. The distance between Line B and Line C (yellow double-headed arrow) is recorded as the osteophyte size. CT: computed tomography.

Using a Picture Archiving and Communication System workstation, the largest sagittal size was measured in the sagittal view of the CT scans. The following standardised technique was employed to ensure accuracy. First, a mid-sagittal section of the knee displaying a clearly visible Blumensaat line was selected. Subsequently, a reference line (Line A; Figure 1a) was drawn on the Blumensaat line, extending from the anterior to the most posterior aspect of the femoral cortex. The sagittal section was medially moved to identify the largest posterior condylar osteophyte. The second line (Line B) was drawn perpendicular to Line A, copied from the mid-sagittal section on the most posterior aspect of the femoral cortex (Figure 1b). Finally, a third line (Line C), parallel to Line B, was drawn on the most posterior edge of the osteophyte. The distance between Lines B and C was recorded as the size of osteophytes. The obtained size measurements were subsequently categorised into four groups based on a classification system – the absence of osteophytes (group A), <5 mm (group B), 5–10 mm (group C), and >10 mm (group D) – to facilitate the comprehensive assessment and analysis of osteophyte size in the study population.

### 2.4. Statistical Analysis

All statistical analyses were performed using SPSS for Windows (version 19.0; IBM Corp., Armonk, NY, USA). Statistical significance was set at *p* < 0.05. All measured values are expressed as the mean ± standard deviation. All data were tested for normal distribution using the Kolmogorov–Smirnov test. If the differences showed a normal distribution, a paired *t*-test was used to compare the gap changes measured using the robotic system before and after bone cutting. In the subgroup analysis of osteophyte size, a one-way analysis of variance for continuous variables was used to compare the four subgroups. In addition, a post hoc Bonferroni test was used to compare pairs of subgroups.

## 3. Results

The demographic characteristics of the study population are presented in Table 1.

Of the 129 knees analysed, 25 knees (19.4%) showed no extension gap changes, and 41 knees (31.8%) showed no flexion gap changes, respectively, after bone cuts (Table 2). Overall, the gaps in medial extension (ME), lateral extension (LE), medial flexion (MF), and lateral flexion (LF) increased significantly after bone cuts (Table 3).

The ME (*p* = 0.001), LE (*p* = 0.001), MF (*p* = 0.002), and LF (*p* = 0.001) gaps increased with the osteophyte size. For osteophytes <10 mm (groups A, B, and C), the increased gaps in ME, LE, MF, and LF were not significantly different, indicating symmetrical gap changes. However, for osteophytes >10 mm (group D), ME, LE, MF, and LF were significantly different, and post hoc analysis showed that the ME gap change was significantly higher than the other gaps, indicating asymmetrical gap changes (Table 4).

## 4. Discussion

Our results show a positive correlation between the size of the unreachable osteophytes of the posterior medial femoral condyle and the extension and flexion gaps. Of all the knees that underwent R-TKA, 80.6% exhibited an increased extension gap after bone cuts, whereas approximately 68.2% showed an increased flexion gap. When the size of the osteophyte was <10 mm, symmetrical increases in both the extension and flexion gaps were observed. In contrast, for osteophyte size exceeding 10 mm, a significant increase in the ME gap was observed compared to the LE, MF, and LF gaps, leading to an asymmetrical mediolateral extension gap.

In this study, all gaps (ME, LE, MF, and LF) increased after bone cutting, and this trend was also observed in the no-osteophytes group. These findings suggest that, regardless of the presence of osteophytes, the routine bone-cutting process leads to changes in the extension and flexion gaps. A prior study conducted by Sugama et al. [14] demonstrated that the initial ME gap measured by a tensioning device after cutting the distal femur and tibia increased by approximately 2.5 mm following the preparation of the flexion gap. Because the final extension gap was determined without a trial implant, and the impact of the femoral implant’s condylar volume was not considered, the gap change was larger than that in our study. Kakuta et al. [15] reported a similar outcome. In this study, the joint gaps were measured at three stages: posterior femoral condylar resection, posterior osteophyte removal, and femoral component placement. This demonstrated the occurrence of a significant increase in the ME gap following femoral bone cutting. Thereafter, the ME gap was reduced by 0.6 mm following femoral component placement. Seo et al. [16] demonstrated similar results, with the bone-cutting process resulting in an increase in the extension gap by 1 mm. We can assume that the adhesion of the posterior capsule and the periarticular ligament structure surrounding the femoral condyle was released after the bone cuts. 

Based on the observation that the size of the medial posterior femoral condyle osteophyte increases, we assumed that the tenting effect of the posterior capsule also increases, resulting in a widening of the gap. Theoretically, only the ME gap should be affected by medial osteophytes; however, in practice, the LE, MF, and LF gaps also increased, which agrees with the results of previous studies. In a study by Baldini et al. [17], the extension and flexion gaps were measured using a tension device, and a symmetrical gap increase was noted in the flexion and extension gaps after posterior condylar osteophyte removal. Sriphirom et al. [18] reported similar results, showing that the presence of a posterior condylar osteophyte in the femur resulted in an increase in both the extension and flexion gaps measured using a computer-assisted system. Unlike our study, these two studies showed that removal caused a greater increase in the flexion gap, while neither of these studies analysed the results according to osteophyte size. Gustke et al. [19] demonstrated the effect of posterior osteophytes on the size and location by measuring gaps using a robot-assisted system. In contrast to previous studies, no significant differences were observed, regardless of the presence of osteophytes.

In our study, for osteophytes <10 mm in size, the ME, LE, MF, and LF gaps increased symmetrically. However, for osteophytes >10 mm, the ME gap increased more asymmetrically than the LE, MF, and LF gaps (*p* < 0.05). Holst et al. [20] conducted a cadaveric study to evaluate the effects of 10 mm and 15 mm 3D-printed osteophyte-mimicking blocks on the medial and lateral contact forces using Verasense (OrthoSensor-Dania Beach, FL, USA). Although there were no significant differences between the 10 mm and 15 mm blocks on the medial contact forces, the presence of blocks caused an asymmetric contact force between the ME and LE. This indicates that the formation of a large osteophyte over time results in asymmetrical tightness of the medial side of the joint. However, if symmetric bone cutting is performed without considering this osteophyte effect, the posterior capsule, tightened by the osteophytes, would loosen again, resulting in an unexpected increase in the asymmetric gap. In cases where the osteophyte size is <10 mm, symmetrical gap changes can be expected, and the insertion of a thicker polyethylene insert can effectively address the issue without additional soft-tissue release and bone-cutting measures; however, when dealing with osteophytes thicker than 10 mm, performing symmetrical gap planning prior to bone cutting may result in an asymmetrical extension gap. Additional bone cuts and soft-tissue release are necessary to achieve a balanced mediolateral extension gap. Moreover, once the lateral extension gap matches the medial extension gap, resolving the mismatch between the extension and flexion gaps becomes a challenge. Therefore, for patients with osteophytes measuring >10 mm, it is advisable to plan for a slightly tighter medial extension gap by reducing the medial distal femoral resection by 2 mm. For patients with osteophytes measuring <10 mm, a symmetrical gap increase of up to 1 mm is negligible, and no additional measures need to be taken.

Our study has several limitations. First, we focused solely on varus osteoarthritic patients. Therefore, the observed changes in joint gaps may vary in valgus knees or varus knees with a predominant lateral femoral osteophyte because of differences in knee structures between the medial and lateral aspects. Second, gap recordings may be subjective. Herein, the extension and flexion medial gaps were measured by applying manual varus and valgus forces to the knee joints; consequently, the recorded values could vary based on the applied stress forces. However, this study was conducted by a single highly experienced surgeon with a high volume of cases, ensuring consistency in the application of stress forces.

## 5. Conclusions

Bony resection resulted in various changes in the flexion and extension gaps linked to the size of the osteophytes of the posterior medial femoral condyle. When patients have posterior osteophytes >10 mm, surgeons should expect an asymmetrical extension gap after bony resection; therefore, a slightly tight medial extension gap should be planned to achieve the desired gaps using the pre-resection gap balancing technique.

## Figures and Tables

**Table 1:** Patient demographics.

Characteristics	Values
Gender (male:female)	97:32
Left:right	79:50
K-L grade (III:IV)	27:102
	Mean ± SD
Age (years)	69.4 ± 5.52
BMI (kg/m^2^)	25.84 ± 3.89
Mean HKA angle (°) ^a^	6.43 ± 2.87
Size of osteophyte on posterior medial femoral condyle (mm)	
Group B	3.25 ± 1.03
Group C	6.93 ± 1.36
Group D	11.96 ± 1.09

^a^ A positive value denotes varus alignment; SD: standard deviation; BMI: body mass index; K-L: Kellgren–Lawrence; HKA: hip–knee–ankle.

**Table 2:** Percentage of gap change following bone cutting.

**Extension Gap Change after Cutting**					
	**Medial Gap Δ**	**≥2 mm**	**1 mm**	**0 mm**	**Number of Knees** **(%)**
**Lateral Gap Δ**					
**≥2 mm**		15 (11.6)	14 (10.9)	0 (0.0)	29 (22.5)
**1 mm**		21 (16.3)	37 (28.7)	9 (7.0)	67 (51.9)
**0 mm**		2 (1.6)	6 (4.7)	25 (19.4)	33 (25.6)
**Number of knees (%)**		38 (29.5)	57 (44.2)	34 (26.4)	129 (100)
**Flexion Gap Change after Cutting**					
	**Medial Gap Δ**	**≥2 mm**	**1 mm**	**0 mm**	**Number of Knees** **(%)**
**Lateral gap Δ**					
**≥2 mm**		6 (4.7)	4 (3.1)	1 (0.8)	11 (8.5)
**1 mm**		9 (7.0)	47 (36.4)	5 (3.9)	61 (47.3)
**0 mm**		4 (3.1)	12 (9.3)	41 (31.8)	57 (44.2)
**Number of knees (%)**		19 (14.7)	63 (48.8)	47 (36.4)	129 (100)

Δ: change.

**Table 3:** Gap changes after bone cutting.

Gap Changes (mm)				
	Gap in Planning	Gap after Cutting	Δ	*p*-Value
	Mean ± SD	Mean ± SD	Mean ± SD	
ME	18.68 ± 0.78	19.77 ± 0.95	1.10 ± 0.85	<0.01 *
LE	18.30 ± 0.76	19.28 ± 0.78	0.98 ± 0.72	<0.01 *
MF	18.93 ± 0.85	19.59 ± 0.91	0.79 ± 0.69	<0.01 *
LF	18.62 ± 0.72	19.42 ± 0.71	0.66 ± 0.68	<0.01 *

* paired *t*-test. Δ: change; SD: standard deviation; ME: medial extension; LE: lateral extension; MF: medial flexion; LF: lateral flexion.

**Table 4:** Gap changes according to size of osteophytes on the posterior medial femoral condyle.

Size of Osteophytes and Effects on Gaps from Initial to Trialing					
Δ	No Osteophytes (Group A) (n = 25) Mean ± SD	<5 mm (Group B) (n = 36) Mean ± SD	5–10 mm (Group C) (n = 51) Mean ± SD	>10 mm (Group D) (n = 17) Mean ± SD	*p*-Value
ME	0.53 ± 0.62	0.71 ± 0.69	1.28 ± 0.81	2.11 ± 0.70	<0.01 *
LE	0.59 ± 0.79	0.71 ± 0.62	1.21 ± 0.64	1.32 ± 0.52	<0.01 *
MF	0.52 ± 0.71	0.50 ± 0.59	0.97 ± 0.67	1.13 ± 0.35	<0.01 *
LF	0.35 ± 0.49	0.42 ± 0.50	0.82 ± 0.75	1.00 ± 0.53	<0.01 *
*p*	ns *	ns *	ns *	<0.01 *	

* One-way analysis of variance; SD: standard deviation; ME: medial extension; LE: lateral extension; MF: medial flexion; LF: lateral flexion; ns: not significant; Δ: change.

## Data Availability

The data presented in this study are available on request from the corresponding author. The data are not publicly available.
